# *Bacillus subtilis *as potential producer for polyhydroxyalkanoates

**DOI:** 10.1186/1475-2859-8-38

**Published:** 2009-07-20

**Authors:** Mamtesh Singh, Sanjay KS Patel, Vipin C Kalia

**Affiliations:** 1 Microbial Biotechnology and Genomics, Institute of Genomics and Integrative Biology (IGIB), CSIR, Delhi University Campus, Mall Road, Delhi-110007, India; 2 Department of Biotechnology, University of Pune, Pune-411007, India

## Abstract

Polyhydroxyalkanoates (PHAs) are biodegradable polymers produced by microbes to overcome environmental stress. Commercial production of PHAs is limited by the high cost of production compared to conventional plastics. Another hindrance is the brittle nature and low strength of polyhydroxybutyrate (PHB), the most widely studied PHA. The needs are to produce PHAs, which have better elastomeric properties suitable for biomedical applications, preferably from inexpensive renewable sources to reduce cost. Certain unique properties of *Bacillus subtilis *such as lack of the toxic lipo-polysaccharides, expression of self-lysing genes on completion of PHA biosynthetic process – for easy and timely recovery, usage of biowastes as feed enable it to compete as potential candidate for commercial production of PHA.

## Background

### The natural bioploymers

Polyhydroxyalkanote(s) (PHAs) are natural biopolymers. Many prokaryotic organisms accumulate PHAs as reserve material when carbon (C) source is available in excess in the environment and there is a limitation of nutrients essential for growth. It serves as a food source, which is mobilized by PHA depolymerase under stressed environmental conditions [1]. Although PHAs may generally account upto 90% of the dry cell weight (DCW) of the microbes [2], however their production on industrial scale is still very costly in comparison to petrochemical-based plastics [3,4]. The other basic drawbacks which hinder their exploitation on industrial scale are the highly crystalline nature and very low strength of poly(3-hydroxybutyrate) (PHB), the most well studied PHA. In contrast to homopolymers of 3HB (PHBs), copolymers of small chain length C3-C5 hydroxy acids (scl-HA) and medium chain length C6-C14 mcl-HA of PHA are more ductile, easier to mold and tough [5]. These copolymers have better film forming and mechanical properties quite similar to low-density polyethylene. These features improve their strength and processability [6,7]. Certain microbes can even produce a natural-synthetic hybrid block copolymer of polyhydroxyoctanoate-diethylene glycol, which results in significant changes in their physiochemical and material characteristics [8,9]. Efforts are thus needed towards improving product quality [10] and efficiency of the recovery process, which will result in optimization of yields. In order to reduce the cost of production, efforts are being made to search [11,12] or develop (genetically engineered) strains [13] capable of producing PHAs from inexpensive renewable sources [5,14,15], or even develop transgenic plants for this purpose [16]. The purpose of this review is primarily to consolidate the features of *Bacillus subtilis *as a potential candidate for producing biopolymers – PHAs.

### PHA biosynthesis and producers

The classical PHB biosynthetic pathway consists of reactions catalyzed by three distinct enzymes. The initial reaction involves condensation of two acetyl-CoA molecules to form acetoacetyl-CoA, which is catalyzed by β-ketothiolase (encoded by *pha*A). The second step is the reduction of acetoacetyl-CoA by an NADPH-dependent acetoacetyl-CoA dehydrogenase (encoded by *pha*B). Lastly, the (R)-3-hydroxybutyryl-CoA monomers are polymerized into P(3HB) by P(3HB) synthase (encoded by *pha*C) [3]. In addition, some PHA producers use secondary pathways: i) methylmalonyl CoA pathway, ii) the de novo fatty acid synthetic pathway and iii) via a five-step metabolic pathway assisted by two stereospecific 2-enoyl-CoA hydratases prior to polymerization [5].

Of the three genes generally reported to be involved in PHA biosynthesis, *phaA *and *phaB *genes are also involved in glyoxylate regeneration in certain α-Proteobacteria and Actinobacterium. In addition, *phaA *gene is also involved in the synthesis of mevalonate, which is ubiquitously present in plants. The transfer of *phaC *gene into an organism gives a new dimension to the functioning of *phaA *and *phaB *genes, which occur together at a much higher frequency in different organisms than with *phaC *gene [17]. PHA synthases belonging to the α/β hydrolase superfamily from 45 different bacteria show an overall identity of 8 to 96%. These could be grouped into 4 different classes [1]: Class I and II PhaC, made up of one subunit (61 to 73 kDa) could produce scl-PHAs (in *Ralstonia eutropha*) and mcl-PHAs (in *Pseudomonas aeruginosa*), respectively. Class III PHA synthases reported from *Allochromatium vinosum *have two types of subunits: i) PhaC and ii) PhaE (approximately 40 kDa each) which preferably synthesize scl-PHAs. Class IV PHA synthases, which resemble Class III PHA synthases (PhaE is replaced by a 20 kDa PhaR) have been reported only in *Bacillus *spp. [1].

A perusal of the capacities of different microbes to produce PHAs reveals that certain Gram-negative bacterial species belonging to *Alcaligenes*, *Ralstonia *and *Pseudomonas *lead this group. *Alcaligenes *and *Ralstonia *are versatile organisms with well established abilities to utilize pure substrates, agricultural wastes, oily wastes, dairy products and carbon dioxide (CO_2_) for PHA production [5,18-20]. *Pseudomonas *can normally synthesize mcl-PHA on various aliphatic alkenes or fatty acids, agricultural and oily wastes [20,21]. *Pseudomonas *sp. can however, simultaneously produce scl-mcl PHAs [22-26]. Among the Gram-negative bacteria, certain archaeal strains of *Haloferax*, *Halobacterium*, *Haloarcula *and *Haloquadratum *have been reported for their abilities to synthesize PHA from inexpensive C sources as feed material [27,28]. Although Gram-positive bacteria have not been widely studied, a few genera reported to produce PHB and certain copolymers include *Bacillus*, *Clostridium*, *Corynebacterium*, *Nocardia*, *Rhodococcus*, *Streptomyces *and *Staphylococcus *[29].

### *Bacillus *as PHA producer

*B. subtilis *is generally regarded as safe (GRAS) organism by Food and Drug Administration (FDA) [30,31] and thus offers additional benefits. *B. subtilis *has been accorded the designation of industrial workhorse for being among the most widely used microbes for large scale production of recombinant proteins, amino acids and fine chemicals [32,33]. It will not be inappropriate to call them as "cell factory" [34] since *B. subtilis *is already known for production of valuable metabolites, bioremediation and generation of bioenergy [35-37] but much attention has not been paid to them as PHA producers. Among the *Bacillus *spp. reported to be PHA producers, the PHA yields vary from 11 to 69% (w/w of DCW – upto 70 g/L): *B. amyloliquefaciens *DSM7, *B. laterosporus*, *B. licheniformis*, *B. macerans*, *B. cereus, B. circulans*, [6], *B. firmus *G2, *B. subtilis *K8,*B. sphaericus *X3, *B. megaterium *Y6 [38], *B. coagulans*, *B. brevis*, *B. sphaericus *ATCC 14577 [39], *B. thuringiensis *[37], *B. mycoides *RLJ B-017 [40] and *Bacillus *sp. JMa5 [41]. *Bacillus *sp. INT005 and *B. cereus *UW85 could produce PHA with a wide range of compositions varying from PHB, P(3HB-co-3HV: copolymer of butyrate and valerate), P(3HB-co-3HHx: copolymer of butyrate and hexanoate), P(3HB-co-4HB-co-3HHx: copolymer of 3-hydroxy-, 4-hydroxy- butyrates and hexanoate) to P(3HB-co-6HHx-co-3HHx: terpolymer of butyrate, 6-hydroxy- and 3-hydroxy hexanoates) depending upon the substrate [42,43]. Various *Bacillus *spp. have been shown by different researchers to synthesize copolymers when co-fed with various substrates. Using *B. cereus *UW85, the production of terpolymer of 3HB, 3HV and 6HHx was recorded with ε-caprolactone as sole C source in mineral salts medium without any glucose. However, addition of glucose along with ε-caprolactone seemed to suppress copolymer synthesis and the result was the production of PHB [42]. *Bacillus *sp. INT005 could accumulate PHB when glucose was used alone as C substrate in the medium. However, addition of various C sources along with very low glucose concentration resulted in copolymers of 3HB and 3HHx on octanoate and decanoate, copolymers of 3HB-4HB-4HHx on 4-hydroxybutanoate and 3HB-3HHx-6HHx on supplementation with ε-caprolactone [44]. Recent studies have produced still more interesting information. *B. cereus *SPV when grown on structurally unrelated C sources such as fructose, sucrose and gluconate resulted in the incorporation of 4HB with the first two substrates and 4HB and 3HV with gluconate in the medium [45]. Although limitation of nitrogen (N), phosphorous (P) and oxygen in the culture conditions are known to influence PHB production, however, potassium limiting media led to the production of a copolymer containing 3HB and 3HV monomers in contrast to only PHB under sulphur, P or N limitation [43]. *B. megaterium *yielded PHB on cane molasses [46], *B. cereus *CFR06 on starch [47] and other *Bacillus *spp. could also produce PHB from industrial food waste water, soya waste and malt waste from beer brewery plant and pea-shell slurry [48]. *Bacillus *sp. 256 utilize an unrefined natural substrate – mahua (*Madhuca *sp.) flower as C source (containing 57% w/w as sugars) to produce copolymers (90:10 mol% P(HB-co-HV)) [49].

There are a few reports which have shown *B. subtilis *to be natural producers [37-39,50]. These results need to be viewed more cautiously since *B. subtilis *is a very heterogeneous group. Recent studies have shown that *B. subtilis *can be subdivided into two subspecies namely *B. subtilis *subsp.*subtilis *and *B. subtilis *subsp.*spizizenii *[51,52], such that only one of them may possess members which are PHA producers, where as those belonging to *B. subtilis *subsp. *subtilis *(*B. subtilis *168 belongs to this group) may be naturally non-producers types. This results in a very interesting situation with respect to *B. subtilis*, which can thus be "labeled" as non-producers just like *Escherichia coli *which has been widely used for expression of many genes including those of PHA biosynthesis [3,5,53-56].

*B. subtilis *has been recently used as a host for over expression of *phaCAB *genes from *P. aeruginosa *and *R. eutropha*. Expression of *phaC1 *from *P. aeruginosa *and *phaAB *genes from *R. eutropha *in *B. subtilis *resulted in the production of copolymers P(HD-co-HDD, hydroxydecanoate-co-hydroxydodecanoate) and P(HB-co-HD-co-HDD) from malt waste [57]. Since *B. subtilis *is not a human pathogen, further supporting its usage as a host for expression of foreign genes [58]. *B. megaterium phaPQRBC *genes were cloned into *B. subtilis *1A304(Φ105MU331). This report of stable plasmid integration into the *B. subtilis *chromosome paved the way for large scale fermentation process for production of PHA which eliminated the use of antibiotics, a desirable feature for reducing production cost [58]. Recombinant *B. subtilis *could utilize malt waste as a C source, further raising the hopes for producing PHA at cheaper rates. This study showed that *phaPQ *of *B. megaterium *were essential for PHA production along with *phaRBC*, although they could not observe any sequence homology in NCBI database. It has been previously shown that *phaP *in *R. eutrophus *might play a role in determining the shape and size of the inclusion bodies [59].

Although *B. subtilis *has the potential to compete with *E. coli*, the most commonly used host for heterologous expression of genes [34] and it may even be superior in certain aspects, however, the time pressure limits the change of host organism in later stages of development of process optimization [60]. Westers *et al*., [60] listed four drawbacks in using *Bacillus *as host organism – a) lack of suitable expression vectors, b) plasmid instability, c) presence of protease and d) presence of malfolded proteins. Recent efforts have circumvented certain issues such as the generation of suitable expression vectors [61] and stable plasmids particularly for PHA production [58]. *B. subtilis *WB800 strain lacking eight extracellular proteases enabled the production of homologous proteins, including those which were otherwise susceptible to rapid degradation [34]. Certain other features which further support *B. subtilis *as potential PHA producer have been discussed here.

### Genomic status of PHA biosynthesis in *Bacillus*

In the post genomic era, 22 *Bacillus *strains have been sequenced and 32 projects are in progress [62]. Among all *Bacillus *spp., *B. subtilis *is one of the most heterogeneous representative [52,63,64]. The concerted efforts of European Union funded consortia [65-67] lead *B. subtilis *[68] to be the first Gram-positive, soil microorganism to be completely sequenced and opened the era of functional analysis of Gram-positive bacteria. The major resources made available as a results of these efforts in *B. subtilis *include datasets on transcriptome, proteome, secretome and metabolome. These also include a collection of more than 3000 mutants, vectors, tools and techniques for rapid production of heterologous proteins [35,69,70].

Screening of metabolic (KEGG) and genomic (NCBI) databases for the presence of enzymes [71] involved in PHA biosynthesis reveals that genes for PhaA and PhaB are observed to be present in almost all the sequenced genomes of *Bacillus *except certain strains of *B. megaterium *[29,72], *B. thuringiensis, B. subtilis *and *Bacillus *spp. (Table 1). However, quite a bit of variation is recorded in the case of *phaC *gene and *phaR*. PhaC is frequently observed largely in the members of *B. cereus *group, however, it's conserved domain is absent or partially present in most other *Bacillus *spp. The presence of conserved domain of PhaR was largely partial or absent. In general *B. subtilis *lacks genes related to PHA biosynthesis [1] providing opportunity to circumvent the need to eliminate or reduce the background effect caused due to homologous genes of the host during heterologous gene expression. The concomitant presence of PHA biosynthesis and depolymerization system has proved beneficial for efficient production of PHA [35]. Incidentally, *B. subtilis *does contain *phaZ *encoding for PHA depolymerase.

**Table 1:** Conserved domains of enzymes for biosynthesis and depolymerization of polyhydroxyalkanoate of *Bacillus *species.

**Description**	**β-Keto thiolase**	**Acetoacetyl reductase**		**Polyhydroxyalkanoate**		
				
				**Synthase**		**Depolymerase**
	
	**PhaA**	**PhaB**		**PhaC**	**PhaR**	**PhaZ**
	
	**Thiolase**	**NADB**	**FabG**	**PhaC**	**PRK** **03918**	**DepA**
*Bacillus cereus *03BB102	F^a^	F	F	F	P^b^	F
*B. cereus *03BB108	F	F	F	F	P	F
*B. cereus *AH1134	F	F	F	F	A^c^	F
*B. cereus *AH187	F	F	F	F	P	F
*B. cereus *AH820	F	F	F	F	P	F
*B. cereus *ATCC 10987	F	F	F	F	P	F
*B. cereus *ATCC 14579	F	F	F	F	A	F
*B. cereus *B4264	F	F	F	F	A	F
*B. cereus *E33L	F	F	F	F	P	F
*B. cereus *G9241	F	F	F	F	P	F
*B. cereus *G9842	F	F	F	F	A	F
*B. cereus *H3081.97	F	F	F	F	P	F
*B. cereus *NVH0597-99	F	F	F	F	P	F
*B. cereus *Q1	F	F	F	F	P	F
*B. cereus *subsp. cytotoxis NVH 391–98	F	F	F	F	A	F
*B. cereus *W	F	F	F	F	P	F
*B. thuringiensis *serovar israelensis ATCC 35646	F	F	F	F	A	F
*B. thuringiensis *serovar konkukian str. 97-27	F	F	F	F	P	F
*B. thuringiensis *str. Al Hakam	F	F	F	F	P	F
*B. anthracis *str. A0193	n/a^d^	F	F	F	P	F
*B. anthracis *str. A0389	n/a	F	F	F	P	F
*B. anthracis *str. A0442	n/a	F	F	F	P	F
*B. anthracis *str. A0465	n/a	F	F	F	P	F
*B. anthracis *str. A0488	n/a	F	F	F	P	F
*B. anthracis *str. A2012	F	F	F	F	n/a	F
*B. anthracis *str. Ames	F	F	F	F	P	F
*B. anthracis *str. 'Ames Ancestor'	F	F	F	F	P	F
*B. anthracis *str. Sterne	F	F	F	F	P	F
*B. coagulans *36D1	F	F	F	A	A	A
*B. coahuilensis *m4-4	F	F	F	F	A	F
*B. weihenstephanensis *KBAB4	F	F	F	F	A	F
*B. amyloliquefaciens *FZB42	F	n/a	n/a	n/a	n/a	n/a
*B. subtilis *subsp. *subtilis *str. JH642	n/a	n/a	n/a	n/a	n/a	n/a
*B. subtilis* subsp. subtilis str. SMY	F	F	F	n/a	n/a	n/a
*B. subtilis *subsp. subtilis str. 168	n/a	F^e^	F^e^	n/a	n/a	n/a
*B. subtilis* subsp. subtilis str. NCIB 3610	F	F	F	A	A	F
*B. licheniformis*	F	F	F	n/a	n/a	n/a
*B. licheniformis *ATCC 14580	F	F	F	n/a	n/a	n/a
*B. pumilus *ATCC 7061	F	F	F	A	A	A
*B. pumilus *SAFR-032	F	F	F	A	A	A
*B. halodurans *C-125	F	F	F	A	A	A
*B. clausii *KSM-K16	F	F	F	A	A	A
*B. megaterium*	n/a	F	F	F	A	F
*B. selenitireducens *MLS10	F	F	F	A	A	A
*Bacillus *sp. B14905	F	F	F	A	A	A
*Bacillus *sp. C18	n/t^f^	n/t	n/t	F	n/t	P
*Bacillus *sp. C19	n/t	n/t	n/t	P	n/t	P
*Bacillus *sp. E13	n/t	n/t	n/t	P	n/t	P
*Bacillus *sp. INT005	na	F	F	F	P	F
*Bacillus *sp. NRRL B-14911	F	F	F	F	A	A
*Bacillus *sp. SG-1	F	F	F	A	A	A

a: Full domain present

b: Partial domain present

c: Domain absent

d: Not applicable

e: Enzyme was β-ketoacyl-acyl carrier protein reductase and the gene was *fabG*

f: Not traceable (due to partial sequencing)

Sequence analysis and pathway alignment of polyhydroxyalkanoate metabolism was done as described earlier [71]. Screening of *Bacillus *spp. in KEGG (Kyoto Encyclopedia of Genes and Genomes)  database was performed for PHA biosynthesis enzymes β-ketoacyl-CoA thiolase (PhaA-EC 2.3.1.9), an NADPH dependent Acetoacetyl-CoA reductase (PhaB-EC 1.1.1.36) and PHA synthase (PhaC-EC 2.3.1.41). The conserved domains for these enzymes were identified from RPS-BLAST [71] (reverse position-specific – basic local alignment search tool) at National Center for Biotechnology Information (NCBI) . Amino acid sequences of PhaA (GenBank accession no. AAP11875), PhaB (GenBank accession no. AAP08300), and PhaC (including PhaZ) (GenBank accession no. AAP08301) of *Bacillus cereus *ATCC 14579, and PhaR (GenBank Accession no.: AAD05258) of *B. megaterium *were used as a queries against the *Bacillus *sequenced genome database using BLAST.

### Genome scale reconstruction

The basic quest in biotechnology is to construct microbial strains capable of accomplishing the rapidly expanding array of desired biotransformations [34]. The initial steps towards enhancing product efficiency and recovery process are to look for the presence of pathways that divert fluxes towards undesirable products or compete for the utilization of precursors and cofactors [73]. Genome scale reconstruction of *B. subtilis *was converted into an *in silico *model to trace the metabolic pathways which can be verified through experimental works in *B. subtilis *[73,74]. This can be executed with relatively high confidence with the availability of databases on transcriptomes, proteomes and metabolomes [73,74]. Biolog's Phenotype Microarray™ technology enables phenotype analysis [74] of *B. subtilis *for a wide range of C, N, P and sulphur sources. A wide variety of C sources and their corresponding transporters present in *B. subtilis *reflected on its high adaptability to environmental conditions. The interesting part was its inability to utilize glycine probably because of production of H_2_O_2_, a toxic byproduct of glycine oxidase activity. The absence of glycoxylate shunt in *B. subtilis *justifies the observation that no growth is observed on acetate as sole C source although NADH and ATP could be produced by the metabolic network [74]. The phenotypes showing the utilization of various substrates are indicative of the presence of corresponding pathways. In the work of Oh *et al*., [74] 350 intracellular metabolites were identified but only 160 were present in the model, which implies that a large portion of *B. subtilis *metabolism is yet to be elucidated.

### Synthetic genomics: a reductionist's approach

The urge to exploit microorganisms for maximum benefits to human beings has emerged in creation of cells with predictable behaviour. Genome reduction process for building collection of knockout mutants has been initiated for a wide range of bacteria such as Gram-positives – *B. subtilis *(Firmicutes), *Corynebacterium glutamicum *(Actinobacteria), and Gram-negatives – *E. coli *K-12, *Haemophilus influenzae*, *P. aeruginosa*, *P. aeruginosa *strain PA14 and *Acinetobacter baylyi *ADP1 (Gamma Proteobacteria) [73]. Efforts in this direction have been made with *E. coli *and *B. subtilis *as model organisms (Table 2), for studying their diverse biological features [32,75]. Since the most daunting task is to predict genes to be deleted without detrimental effect(s), Hoshimoto *et al*., began with constructing and characterizing a series of large scale chromosomal deletion mutants of *E. coli *[76]. The 16 mutants of *E. coli *K-12 strain MG1655 lacked 2.4–29.7% of the parental chromosome. These deletions resulted in aberrant cell shape, slow growth rate and modified nucleoid organization. Mutants of *E. coli *MG1655 and W3110 with the removal of mobile DNA, virulence genes and genes for amino acids, lipopolysaccharides, transporters etc. exhibited high electroporation efficiency [77] and higher threonine secretion [75] compared to wild type strain. *B. subtilis *the Gram-positive counter-part of the industrially important *E. coli *has only 271 essential genes out of a 4.2 Mb genome [78]. The optimization of *Bacillus *as cell factory by deleting prophages, prophage-like regions and polyketide synthase operon of *B. subtilis *amounted to 7.7% reduction in genome size equivalent to elimination of 332 protein encoding genes [32]. Subsequent efforts by Ara *et al*., 2007 lead to DNA deletion to the extent of 24.7% without any apparent benefits [79]. However, Morimoto *et al*., 2008 [61] have demonstrated that genome reduction may create bacterial cells with practical utility to industry – enhanced extracellular cellulase and protease and improved efficiency to utilize a wide range of C sources.

**Table 2:** Unique features of reduced genomes of *Escherichia coli *and *Bacillus subtilis*

**Organism**	**Parent strain**	**Mutant strain**	**Reduction in genome size**		**DNA removed**	**Unique characteristics**	**Ref**
						
			**Mb**	**%**			
*Escherichia coli *K-12	MG1655	Δ16	1.38	29.7	Large scale deletions	Aberrant cell morphology Increased doubling time Changed nucleoid organization	[76]

*E. coli *K-12	MG1655	MDS42	0.71	15	Mobile DNA, Cryptic virulence genes	Normal cell growth and protein expression, comparable to parental strain MDS42 cells exhibit high electroporation efficiency and propagation of unstable plasmid	[77]

*E. coli *K-12	W3110	MGF-01	1.03	22	Biosynthesis genes for some amino acids, lipopolysaccharides and phosphor lipids Transporter genes, ISs and toxin-antitoxin pairs	Growth was as rapid as the parental strain in minimal medium (M9) during exponential phase. Mutant strain continued whereas wild type strain progressed to the stationary phase MGF-01 secreted higher (2.44-fold) threonine compared to wild type strain	[75]

*Bacillus subtilis*	168	Δ6	0.32	7.7	Prophages – SPβ, PBSX Prophage like sequences – *pro1*, *pro3*, *skin *(*sigK *intervening) *pks *operon	No unique properties including AmyQ protein secretion Increase in heterologous amylase secretion	[32]

*B. subtilis*	168	MGB469	0.50	12.5	All prophages All prophage like sequences except *pro7* *pks *and *pps *operons	Cell growth was normal No beneficial properties were apparent	[79]

*B. subtilis*	MGB469	MG1M	0.99^a^	24.7	All prophage All prophage like sequences except *pro7* *pks*, *pps *operon and 6 deletions	Unstable phenotypes with regard to growth rate, cell morphology and recombinant protein production	[79]

*B. subtilis*	MGB469	MGB874	0.87^a^	20.7	Eleven non essential gene cluster i.e. 865genes Genes essential for spore formation including *spoIIIC *and *spoIVCB*	Enhanced productivity of extracellular cellulase and protease Reduced growth rate (30% in LB, 50% in SMM compared with wild type *B. subtilis *168 strain Did not form spores Improved efficiency of carbon source utilisation	[61]

a: with respect to *B. subtilis *168 genome

*B. subtilis *Δ6 has a few major advantages compared to conventional *B. subtilis *strains. *B. subtilis *Δ6 lacks the BsuM restriction-modification system. It confers advantages to Δ6, since BsuM restriction reduces transformation efficiency of *B. subtilis *with recombinant plasmids and is also responsible for structural plasmid instability. It consequently affects the application potential of plasmids for high-level protein production [32]. This system can be assisted by highly stable expression vectors developed for the rapid purification of recombinant protein in *B. subtilis *[80]. However, this cell factory engineering can be used to re-insert genes of importance into the chromosome of *B. subtilis*.

### Horizontal Gene Transfer (HGT)

HGT plays an important role in the diversity and evolution of *B. subtilis*. Phage integration accounts for 16% of the HGT regions in *B. subtilis *168 [81]. Extensive HGT in *Bacillus *sp. increases the chance of "easier" and extensive manipulation. HGT from A+T rich islands can be accomplished by transposes. *B. subtilis *has comparatively more stable chromosomal structure compared to other *Bacillus *spp. particularly those of *B. cereus *group. *B. subtilis *genome shows 10 transposons and related rearrangements compared to 112 such events in *B. halodurans *C-125 chromosome [82]. In *B. subtilis *the ability to survive in a wide range of environmental niches can be because of large number of genes (586 equivalent to 10% of the ORFs) related to SpoA, regulatory protein important for initiating sporulation, genes encoding proteins involving antibiotics and cell wall synthesis [63].

### Manipulating carbon catabolite repression

In view of the fact that feedstock being a significant component of manufacturing costs of PHA, maximizing the C yield and reduced byproduct formation becomes a priority. The metabolic bottleneck for maximum yield of all products is either stoichiometrically constrained or limited by energy. Sauer *et al*., [83] suggested that to improve yields, it will be better to look for microorganisms and processes that may provide the necessary energetic efficiency [83]. They recommended either to use alternative C substrates such as glycerol or sucrose (instead of glucose) which have reduced or non-existing stoichiometric limitations or go in for metabolic engineering [83]. For maximum efficiency and regulation of metabolic processes in *B. subtilis*, an alternative C catabolite repression was suggested since it doesn't possess cAMP [84]. A CcpA defective mutant of *B. subtilis *was found to grow at a slower rate in minimal medium with glucose and ammonia as C and N sources, in comparison to the wild type cells [84]. CcpA represses the expression of *citZ *gene encoding citrate synthase. This reaction involves condensation (utilization) of acetyl-CoA with oxaloacetate [84]. Manipulation of CcpA can enable larger availability of acetyl-CoA for PHA biosynthesis. Further, *B. subtilis *sporulates in response to depletion of nutrients [64] and glucose [79]. Incidentally, there is a conflict between PHA production and spore formation, since both get induced under similar environmental conditions. A non-spore forming strain (wild or mutant) of *Bacillus *holds promise of better performance [2]. So a knockout will enable it to grow vegetative and make sporulation genes redundant. In fact, this mechanism lead to increased cellulase activity in *B. subtilis *168 [79]. Under laboratory conditions, it will be worth the effort to knockout sporulations genes and make room for re-introducing relevant genes, if necessary. Gram-positive bacteria can produce copolymers of PHA from simple, inexpensive and structurally un-related C sources. In contrast, Gram-negative bacteria depend upon expensive and structurally related substrates for producing copolymers of PHA [29].

### Novel recovery system for PHA

Since PHA accumulates intracellularly, cell disruption is indispensable to recover it. On an industrial scale, the PHA recovery process involves the usage of a large amount of chemical reagents and/or enzymes [2]. An efficient recovery system implies reduced cost of production [85]. An optimum time to recover PHAs is immediately after the C source gets exhausted and before the commencement of degradation by depolymerase enzyme [86]. A novel self disruption cell system has been developed in *B. megaterium*. In this system, a gene cassette carrying the cell lysis system (holin and endolysin of *B. amyloliquefaciens *phage) [87] was inserted into the *E. coli *– *B. subtilis *shuttle vector pX. In this expression system, *xylR*-*xylA' *target genes are induced by xylose but inhibited by glucose, which acts as an anti-inducer [86]. It synchronizes the processes of spontaneous cell lysis and substrate exhaustion, which thus results in the release of accumulated PHAs [86]. The efficiency of this regulatory process can be enhanced by manipulating the YoeB, a cell wall-associated protein, which gets induced in response to antibiotics stress. The expression of *yoeB *in *B. subtilis *is under a xylose-inducible promoter [88]. *yoeB *mutants display an increased rate of autolysis in response to nutrient depletion and various cell envelope stress conditions [88]. The process of autolysis in *B. subtilis *168 can be aided by mutating *yoeB *gene and in the process making it independent of xylose regulation.

### Lacks Lipopolysaccharides

Gram-negative bacteria such as *Ralstonia*, *A. latus *and recombinant *E. coli *are among those which have been exploited for industrial scale PHB production. The outer cell membrane of most Gram-negative bacteria including *E. coli *contains lipo-polysaccharides (LPS), which are endotoxins [89] and are pryogenic in human beings [60]. The purification of byproducts including PHAs is complicated due to the presence of endotoxins [60]. Since LPS induces a strong immunogenic reaction, this feature is undesirable for biomedical applications of the PHAs [7,90]. A review on the toxic nature of LPS reveals that cyanobacterial (Gram-positive bacteria) LPS are less toxic compared to those produced by members of Enterobacteraceae [89]. *B. subtilis *offers the advantage of lacking LPS and excreting proteins at a high rate into the medium [61]. It thus stands a better chance as PHA producer for biomedical applications [90].

### Potential for producing hydrogen

The use of CO_2 _as a potential inexpensive renewable C source can help in reducing PHA production cost [5]. *Synechococcus *sp. MA19 was observed to produce 55% w/w PHA from CO_2 _[91]. *A. eutrophus *was shown to accumulate PHA at the rate of 1.55 g/L/h, which was higher than that recorded with cyanobacteria or photosynthetic bacteria [92]. The strategy being proposed is that if hydrogen (H_2_) production becomes cheap then *R. eutropha *can produce PHB from CO_2 _and oxidation of (H_2_), under dark fermentative conditions [93]. *Bacillus *seems to meet the requirements of this proposal. *Bacillus *spp. such as *B. coagulans, B. licheniformis *and *B. subtilis *have been shown to evolve 1.5 to 2.36 mol H_2_/mol glucose [36]. Biowastes rich in starch such as damaged wheat grains have been employed as feed for generating H_2 _(45 to 64 L/kg Total solids) by *B. licheniformis *and *B. subtilis *[94]. More recently, *B. cereus *strain EGU43 and *B. thuringiensis *strain EGU45 have been reported to generate 0.63 mol of H_2_/mol of glucose and upto 500 mg PHB/L [37].

## Conclusion

*B. subtilis *as an organism for industrial scale production of several fine chemicals is already established but not for PHA production. A comparison of all the PHA producers (Additional file 1, Table S1) reveals that the most versatile PHA producers are *Bacillus *spp. Their abilities to produce PHAs range from homopolymers to copolymers fromsimple sugars to complex industrial wastes. It reflects on how *Bacillus *can exploit its natural abilities to produce hydrolytic enzymes for easy metabolism of biowastes to be used as inexpensive C source. *Bacillus *can be easily grown to very high cell density of 70 g/L [41] and does not have a major codon bias [29,58]. A recovery of around 70 to 80% of bacterial dry matter as PHA production is potentially sufficient for establishing an economically feasible process [35]. In addition, the robust stress tolerant *B. subtilis *lacking the toxic LPS, and permitting heterologous expression of self-lysing genes concomitant with completion of PHA biosynthetic process (for efficient recovery), enable it to be a strong contender in the future as an industrial PHA producer.

## Competing interests

The authors declare that they have no competing interests.

## Authors' contributions

MS and SKSP have contributed towards collection of material, data-mining and preparation of article. VCK has contributed towards the conceptualization and writing the article.

## Supplementary Material

Additional file 1 **Microorganisms producing homopolymers and copolymers of polyhydroxyalkanoates from pure substrates and biowastes**. The data provided represent the microorganisms belonging to different taxonomic groups. Click here for file
