# Supplementary material for: Bacillus subtilis as potential producer for polyhydroxyalkanoates
Source: Microb Cell Fact. 2009 Jul 20;8:38. doi: 10.1186/1475-2859-8-38 (PMC2719590; doi:10.1186/1475-2859-8-38)
Supplement: Additional file 1 — Microorganisms producing homopolymers and copolymers of polyhydroxyalkanoates from pure substrates and biowastes. The data provided represent the microorganisms belonging to different taxonomic groups. [file 1475-2859-8-38-S1.doc]

**Supplementary Table S1: Microorganisms producing homopolymers and copolymers of polyhydroxyalkanoates from pure substrates and biowastes**

| **Substrate** | **Polyhydroxyalkanoate (PHA)** | | | | | | | |
| --- | --- | --- | --- | --- | --- | --- | --- | --- |
|  | **Homopolymers** | | | | **Copolymers** | | | |
|  | **Gram-Positive** | **Ref** | **Gram-Negative** | **Ref** | **Gram-Positive** | **Ref** | **Gram-Negative** | **Ref** |
| **Glucose** | ***Bacillus***  *Streptococcus*  *Streptomyces* | [1-3]  [4]  [5] | *Azotobacter*  *Comamonas*  *Escherichia* ^a^  *Pseudomonas*  *Ralstonia*  *Vibrio* | [12]  [13]  [14]  [15]  [16]  [17] | ***Bacillus*** | [44,45] | *Pseudomonas*  *Ralstonia* | [47] [16,48] |
| **Fructose** | ***Bacillus*** | [3] | *Comamonas*  *Ralstonia* | [13]  [18] | ***Bacillus***  *Microlunatus* | [1]  [46] | *Comamonas* | [13] |
| **Sucrose** | ***Bacillus***  *Streptococcus* | [3,5,6]  [4] | *Alcaligenes*  *Comamonas*  *Vibrio* | [19]  [13]  [17] | ***Bacillus*** | [1] | *Rhizobium*  *Sphingomonas* | [6]  [6] |
| **Lactose** | *Lactobacillus*  *Lactococcus*  *Streptococcus* | [4]  [4]  [4] | *Comamonas*  *Hydrogenophaga*  *Methylobacterium*  *Paracoccus*  *Pseudomonas*  *Sinorhizobium* | [13]  [20]  [21]  [20]  [18]  [20] |  |  |  |  |
| **Fatty acids** | ***Bacillus*** | [1,5] | *Brachymonas*  *Comamonas*  *Pseudomonas*  *Spirulina*  *Vibrio* | [19] [13,22] [23]  [24]  [17] | ***Bacillus***  *Microlunatus* | [1,44] [46] | *Aeromonas* ^a^  *Comamonas*  *Escherichia* ^a^  *Pseudomonas* | [49]  [13]  [50] [51,52] |
| **Maltose** |  |  | *Comamonas*  *Protomonas* | [13]  [25] |  |  |  |  |
| **Methanol** |  |  | *Pseudomonas* | [18] |  |  |  |  |
| **Starch** |  |  | *Azotobacter*  *Haloferax* | [26]  [27] |  |  |  |  |
| **Glycerol** |  |  | *Escherichia* ^a^  *Methylobacterium*  *Ralstonia*  *Vibrio* | [28]  [29]  [29]  [17] |  |  |  |  |
| **Xylose** |  |  | *Burkholderia*  *Methylobacterium* | [30]  [31] |  |  |  |  |
| **Agricultural Waste** | ***Bacillus***  *Staphylococcus* | [3,5,7,8] [9] | *Alcaligenes*  *Azotobacter*  *Burkholderia*  *Escherichia* ^a^  *Haloferax*  *Klebsiella* ^a^  *Ralstonia* | [9,19]  [12]  [30]  [32]  [33]  [34] [35,36] | ***Bacillus*** | [6,45] | *Haloferax Klebsiella*  *Pseudomonas*  *Rhizobium*  *Sphingomonas* | [33]  [9] [43,53] [6]  [6] |
| **Dairy Products** |  |  | *Escherichia* ^a^ *Hydrogenophaga*  *Methylobacterium*  *Pseudomonas*  *Sinorhizobium* | [32,37,38] [20]  [21]  [39]  [20] |  |  | *Pseudomonas*  *Ralstonia* | [39]  [54] |
| **Oily Waste** |  |  | Ralstonia | [40] |  |  | *Comamonas*  *Pseudomonas*  *Ralstonia* ^a^ | [55]  [56]  [40, 57] |
| **Industrial Waste** | *Actinobacillus*  ***Bacillus***  *Rhodococcus* | [10]  [8]  [11] | *Azotobacter*  *Burkholderia*  *Pseudomonas* | [41]  [42]  [43] |  |  | *Azotobacter* | [41] |

a: Recombinant strains

**References**

1. Valappil SP, Misra SK, Boccaccini AR, Keshavarz T, Bucke C, Roy I: **Large-scale production and efficient recovery of PHB with desirable material properties from the newly characterized *Bacillus cereus* SPV.** *J Biotechnol* 2007, **132:**251-258.
2. Porwal S, Kumar T, Lal S, Rani A, Kumar S, Cheema S, Purohit, HJ, Sharma R, Patel SKS, Kalia VC: **Hydrogen and polyhydroxybutyrate producing abilities of microbes from diverse habitats by dark fermentative process.** *Biores Technol* 2008, **99:**5444-5451.
3. Kumar T, Singh M, Purohit HJ, Kalia VC: **Potential of *Bacillus* sp. to produce polyhydroxybutyrate from biowaste.** *J Appl Microbiol* 2009, **106:**2017-2023.
4. Yuksekdag ZN, Beyatli Y: **Production of Poly-beta-hydroxybutyrate (PHB) in different media by *Streptococcus thermophilus* Ba21S strain.** *J Appl Biol Scie* **2:**07-10.
5. Valappil SP, Boccaccini AR, Bucke C, Roy I: **Polyhydroxyalkanoates in Gram-positive bacteria: insight from the genera *Bacillus* and *Streptomyces*.** *Antonie van Leeuwenhoek* 2007, **91:**1-17.
6. Anil-Kumar PK, Shamla TR, Kshama L, Prakash MH, Joshi GJ, Chandrashekar A, Kumari KSL, Divyashree MS: **Bacterial synthesis of poly(hydroxybutyrate-co-hydroxyvalerate) using carbohydrate-rich mahua (*Madhuca* sp.) flowers.** *J Appl Microbiol* 2007, **103:**204-209.
7. Wu Q, Huang H, Hu G, Chen J, Ho KP, Chen GQ: **Production of poly-3-hydroxybutyrate by *Bacillus* sp. JMa5 cultivated in molasses media.** *Antonie van Leeuwenhoek* 2001, **80:**111-118.
8. Vijayendra SVN, Rastogi NK, Shamala TR, Anil-Kumar PK, Kshama L, Joshi GJ: [**Optimization of polyhydroxybutyrate production by *Bacillus* sp CFR 256 with corn steep liquor as a nitrogen source**](http://dx.doi.org/10.1007/s12088-007-0033-7)**.** *Ind* *J Microbiol* 2007, **47:**170-175.
9. Wang YJ, Hua FL, Tseng YF, Chan SY, Sin SN, Chua H, Yu PHF, Ren NQ: **Synthesis of PHAs from waste water under various C:N ratios.** *Biores* *Technol* 2007, **98:**1690-1693.
10. Son H, Park G, Lee, S: **Growth-associated production of poly-β-hydroxybutyrate from glucose or alcoholic distillery wastewater by *Actinobacillus* sp. EL-9.** [*Biotechnol Lett*](http://www.springerlink.com/content/100138/?p=129ed4ccdeb74292ac9052dcda1f045e&pi=0) 2004, **18:**1229-1234.
11. Fuchtenbusch B, Steinbuchel A: **Biosynthesis of polyhydroxyalkanoates from low-rank coal liquefaction products by *Pseudomonas oleovorans* and *Rhodococcus ruber*.** *Appl* *Microbiol* *Biotechnol* 1999, **52:**91-95.
12. Page WJ, Cornish A: **Growth of *Azotobacter vinelandii* UWD in fish peptone medium and simplified extraction of poly-β-hydroxybutyrate.** *Appl Environ Microbiol* 1993, **59:**4236-4244.
13. Lee WH, Azizan MNM, Sudesh K: **Effects of culture conditions on the composition of poly(3-hydroxybutyrate-co-4-hydroxybutyrate) synthesized by *Comamonas acidovorans*.** *Polym* *Degrad Stab* 2004, **84:**129-134.
14. Nikel PI, Pettinari MJ, Galvagno MA, Méndez BS: **Poly(3-hydroxybutyrate) synthesis by recombinant *Escherichia coli* *arcA* mutants in microaerobiosis.** *Appl* *Environ* *Microbiol* 2006, **72:**2614-2620.
15. Bertrand JL, Ramsay BA, Ramsay JA, Chavarie C: **Biosynthesis of poly-β-hydroxyalkanoates from pentoses by *Pseudomonas pseudovora*.** *Appl Environ Microbiol* 1990, **56:**3133-3138.
16. Nurbas M, Kutsal T: **Production of PHB and P(HB-co-HV) biopolymers by using *Alcaligenes eutrophus*.** *Iran Polym* *J* 2004, **13:**45-51.
17. Chien CC, Chen CC, Choi MH, Kung SS, Wei YHC: **Production of poly-β-hydroxybutyrate (PHB) by *Vibrio* spp. isolated from marine environment.** *J* *Biotechnol* 2007, **132:**259-263.
18. Young FK, Kastner JR, May SW: **Microbial production of poly-β-hydroxybutyric acid from D-xylose and lactose by *Pseudomonas cepacia*.** *Appl Environ Microbiol* 1994, **60:**4195-4198.
19. Shi HP, Lee CM, Ma WH: **Influence of electron acceptor, carbon, nitrogen, and phosphorus on polyhydroxyalkanoate (PHA) production by *Brachymonas* sp. P12.** *World J Microbiol Biotechnol* 2007, **23:**625-632.
20. Povolo S, Casella S: **Bacterial production of PHA from lactose and cheese whey permeate.** *Macromol Symp* 2003, **197:**1-9.
21. Yellore V, Desai A: **Production of poly-3-hydroxybutyrate from lactose and whey by *Methylobacterium* sp. ZP24.** *Lett* *Appl* *Microbiol* 1998, **26:**391-397.
22. Zakaria MR, Abd-Aziz S, Ariffin H, Rahman NAA, Yee PL, Hassan MA: ***Comamonas* sp. EB172 isolated from digester treating palm oil mill effluent as potential polyhydroxyalkanoate (PHA) producer.** *Afr J Biotechnol* 2008, **7:**4118-4121.
23. Ashby RD, Solaiman DKY, Foglia TA: **The synthesis of short- and medium-chain-length poly(hydroxyalkanoate) mixture from glucose- or alkanoic acid-grown *Pseudomonas oleovorans*.** *J Ind Microbiol* *Biotechnol* 2002, **28:**147-153.
24. Jau MH, Yew SP, Toh PS, Chong AS, Chu WL, Phang SM, Najimudin N, Sudesh K: **Biosynthesis and mobilization of poly(3-hydroxybutyrate) P(3HB) by *Spirulina platensis*.** *Int* *J* *Biol* *Macromol* 2005, **36:**144-151.
25. Suzuki T, Yamane T, Shimizu S: **Mass production of poly-β-hydroxybutyric acid by fed-batch culture with controlled carbon/nitrogen feeding.** *Appl* *Microbiol* *Biotechnol* 1986, **24:**370-374.
26. Kim BS, Chang HN: **Production of poly(3-hydroxybutyrate) from starch by *Azotobacter chroococcum*.** *Biotechnol Lett* 1998, **20:**109-112.
27. Lillo JG, Rodriguez-Valera F: **Effect of culture conditions on poly(β-hydroxybutyric acid) production by *Haloferax mediterranei*.** *Appl* *Microbiol* **56:**2517-2521.
28. Nikel PI, Pettinari MJ, Galvagno MA, Méndez BS: **Poly(3-hydroxybutyrate) synthesis from glycerol by recombinant *Escherichia coli* *arc*A mutant in fed-batch microaerobic cultures.** *Appl Microbiol* *Biotechnol* 2008, **77:**1337-1343.
29. Bormann EJ, Roth M: **The production of polyhydroxybutyrate by *Methylobacterium rhodesianum* and *Ralstonia eutropha* in media containing glycerol and casein hydrolysates.** *Biotechnol Lett* 1999, **21:**1059-1063.
30. Silva LF, [Taciro MK](http://www.ncbi.nlm.nih.gov/sites/entrez?Db=pubmed&Cmd=Search&Term="Taciro MK"%5BAuthor%5D&itool=EntrezSystem2.PEntrez.Pubmed.Pubmed_ResultsPanel.Pubmed_DiscoveryPanel.Pubmed_RVAbstractPlus), [Ramos ME](http://www.ncbi.nlm.nih.gov/sites/entrez?Db=pubmed&Cmd=Search&Term="Michelin Ramos ME"%5BAuthor%5D&itool=EntrezSystem2.PEntrez.Pubmed.Pubmed_ResultsPanel.Pubmed_DiscoveryPanel.Pubmed_RVAbstractPlus)M, [Carter JM](http://www.ncbi.nlm.nih.gov/sites/entrez?Db=pubmed&Cmd=Search&Term="Carter JM"%5BAuthor%5D&itool=EntrezSystem2.PEntrez.Pubmed.Pubmed_ResultsPanel.Pubmed_DiscoveryPanel.Pubmed_RVAbstractPlus), [Pradella JG](http://www.ncbi.nlm.nih.gov/sites/entrez?Db=pubmed&Cmd=Search&Term="Pradella JG"%5BAuthor%5D&itool=EntrezSystem2.PEntrez.Pubmed.Pubmed_ResultsPanel.Pubmed_DiscoveryPanel.Pubmed_RVAbstractPlus), [Gomez JG](http://www.ncbi.nlm.nih.gov/sites/entrez?Db=pubmed&Cmd=Search&Term="Gomez JG"%5BAuthor%5D&itool=EntrezSystem2.PEntrez.Pubmed.Pubmed_ResultsPanel.Pubmed_DiscoveryPanel.Pubmed_RVAbstractPlus): **Poly-3-hydroxybutyrate (P3HB) production by bacteria from xylose, glucose and sugarcane bagasse hydrolysate.** [*J Ind Microbiol Biotechnol*](http://www.springerlink.com/content/100967/?p=1fb6add6eab946d284d55b5a55002507&pi=0)2004, **31:**245-254.
31. Kim SW, Kim P, Lee HS, Kim JH: **High production of poly-β-hydroxybutyrate (PHB) from *Methylobacterium organophilum* under potassium limitation.** *Biotechnol* *Lett* 1996, **18:**25-30.
32. Liu F, Li W, Ridgway D, Gu T, Shen Z: **Production of poly-β-hydroxybutyrate on molasses by recombinant *Escherichia coli*.** *Biotechnol Lett* 1998, **20:**345-348.
33. Huang TY, Duan KJ, Huang SY, Chen CW: **Production of polyhydroxybutyrates from inexpensive extruded rice bran and starch by *Haloferax mediterranei*.** *J Ind Microbiol Biotechnol* 2006, **33:**701-706.
34. Zhang H, Obias V, Gonyer K, Dennis D: **Production of polyhydroxyalkanoates in sucrose-utilizing recombinant *Escherichia coli* and *Klebsiella* strains.** *Appl* *Environ* *Microbiol* 1994, **60:**1198-1205.
35. Zhang S, Norrlow O, Wawrzynczyk J, Dey ES: **Poly(3-hydroxybutyrate) biosynthesis in the biofilm of *Alcaligenes eutrophus*, using glucose enzymatically released from pulp fiber sludge.** *Appl Environ Microbiol* 2004, **70:**6776-6782.
36. Haas R, Jin B, Zepf FT: **Production of poly(3-hydroxybutyrate) from waste potato starch.** *Biosci Biotechnol Biochem* 2008, **72:**253-256.
37. Park SJ, Park JP, Lee SY: **Production of poly(3-hydroxybutyrate) from whey by fed-batch culture of recombinant *Escherichia coli* in a pilot-scale fermenter.** *Biotechnol Lett* 2002, **24:**185-189.
38. Nikel PI, de Almida A, Melillo EC, Miguel AG, Pettinari MJ: **New recombinant *Escherichia coli* strain tailored for the production of poly(3-hydroxybutyrate) from agro industrial by-products.** *Appl Environ Microbiol* 2006, **72:**3949-3954.
39. Jiang Y, Song X, Gong L, Li P, Dai C, Shao W: **High poly(β-hydroxybutyrate) production by *Pseudomonas fluorescens* A2a5 from inexpensive substrates.** *Enzyme Microb* *Technol* 2008, **42:**167-172.
40. Kahar P, Tsuge T, Taguchi K, Doi Y: **High yield production of polyhydroxyalkanoates from soybean oil by *Ralstonia eutropha* and its recombinant strain.** *Polym Degrad* *Stab* 2004, 83:79-86.
41. Cho KS, Ryu HW, Park CH, Goodrich PR: **Poly(hydroxybutyrate-co-hydroxyvalerate) from swine waste liquor by *Azotobacter vinelandii* UWD.** *Biotechnol Lett* 1997, **19:**7-10.
42. Alias Z, Tan KPI: **Isolation of palm oil-utilising, polyhydroxyalkanoate (PHA)-producing bacteria by an enrichment technique.** *Biores* *Technol* 2005, **96:**1229-1234.
43. Koller M, Bona R, Chiellini E, Fernandes EG, Horvat P, Kutschera C, Hesse P, Braunegg G: **Polyhydroxyalkanoate production from whey by *Pseudomonas hydrogenovora*.** *Biores* *Technol* 2008, **99:**4854-4863.
44. Tajima K, Igari T, Nishimura D, Nakamura M, Satoh Y, Munekata M: **Isolation and characterization of *Bacillus* sp. INT005 accumulating polyhydroxyalkanoate (PHA) from gas field soil.** *J Biosci Bioeng* 2003, **95:**77-81.
45. Wang Y, Ruan L, Chua H, Yu PHF: **Cloning and expression of the PHA synthase genes *phaC1* and *phaC1AB* into *Bacillus subtilis*.** *World J Microbiol Biotechnol* 2006, **22:**559-563.
46. Akar A, Akkaya EU, Yesiladali SK, Celikyilmaz G, Cokgor EU, Tamerler C, Orhon D, Cakar ZP: **Accumulation of polyhydroxyalkanoates by *Microlunatus phosphovorus* under various growth conditions.** *J Ind Microbiol* *Biotechnol* 2006, **33:**215-220.
47. Diniz SC, Taciro MK, Gomez JG, da Cruz Pradella JG: **High cell-density cultivation of *Pseudomonas putida* IPT 046 and medium-chain-length polyhydroxyalkanoate production from sugarcane carbohydrates.** *Appl* *Biochem* *Biotechnol* 2004, **119:**51-70.
48. Yu ST, Lin CC, Too JR: **PHBV production by *Ralstonia eutropha* in continuous stirred tank reactor.** *Proce*ss *Biochem* 2005, **40:**2729-2734.
49. Chien CC, Ho LY: **Polyhydroxyalkanoates production from carbohydrates by a genetic recombinant *Aeromonas* sp.** *Lett Appl Microbiol* 2008, **47:**587-593.
50. Park SJ, Ahn WS, Green PR, Lee SY: **Production of poly(3-hydroxybutyrate-co-3-hydroxyhexanoate) by metabolically engineered *Escherichia coli* strains.** *Biomacromolecules* 2001, **2:**248-254.
51. Kim BS: **Production of medium chain length polyhydroxyalkanoates by fed-batch culture of *Pseudomonas oleovorans*.** *Biotechnol* *Lett* 2002, **24:**125-130.
52. Hartmann R, Hany R, Pletscher E, Ritter A, Witholt B, Zinn M: **Tailor-made olefinic medium-chain-length poly[(R)-3-hydroxyalkanoates] by *Pseudomonas putida* GPo1: Batch versus chemostat production.** *Biotechnol Bioeng* 2006, **93:**737-746.
53. Solaiman DKY, Ashby RD, Jr. Hotchkiss AT, Foglia TA: **Biosynthesis of medium-chain-length poly(hydroxyalkanoates) from soy molasses.** *Biotechnol* *Lett* 2006, **28:**157-162.
54. Marangoni C, Furigo A, Jr. de Aragao MF: **Production of poly(3-hydroxybutyrate-co-3-hydroxyvalerate) by *Ralstonia eutropha* in whey and inverted sugar with propionic acid feeding.** *Process* *Biochem* 2002, **38:**137-141.
55. Thakor N, Trivedi U, Patel KC: **Biosynthesis of medium chain length poly-3-hydroxyalkanoates (mcl-PHAs) by *Comamonas testosteroni* during cultivation on vegetable oils.** *Biores Technol* 2005, **96:**1843-1850.
56. He W, Tian W, Zhang G, Chen GQ, Zhang Z: **Production of novel polyhydroxyalkanoates by *Pseudomonas stutzeri* 1317 from glucose and soybean oil.** *FEMS Microbiol Lett* 1998, **169:**45-49.
57. Bhubalan K, Lee WH, Loo CY, Yamamoto T, Tsuge T, Doi Y, Sudesh K: **Controlled biosynthesis and characterization of poly(3-hydroxybutyrate-co-3-hydroxyvalerate-co-3-hydroxyhexanoate) from mixtures of palm kernel oil and 3HV-precursors.** *Polym Degrad Stab* 2008, **93:**17-23.
